# Association of Hearing Loss With Dementia

**DOI:** 10.1001/jamanetworkopen.2019.8112

**Published:** 2019-07-31

**Authors:** Chin-Mei Liu, Charles Tzu-Chi Lee

**Affiliations:** 1 Department of Health Promotion and Health Education, National Taiwan Normal University, Taipei, Taiwan; 2 Taiwan Centers for Disease Control, Taipei, Taiwan

## Abstract

**Importance:**

Hearing loss (HL) may be a modifiable risk factor for dementia, and longitudinal studies are needed to examine the association of HL and dementia.

**Objective:**

To investigate the association of HL with incident dementia in Taiwanese adults in the general population.

**Design, Setting, and Participants:**

This population-based cohort study collected data from the National Health Insurance Research Database of Taiwan. Patients newly diagnosed with HL from January 1, 2000, through December 31, 2011 (n = 8135), constituted the exposed (HL) group. The HL group patients were matched by sex, age, residence, and insurance premium to individuals without HL (non-HL group) (n = 8135). Data were analyzed from January 1, 2000, to December 31, 2013.

**Exposure:**

Hearing loss defined according to *International Classification of Diseases, Ninth Revision, Clinical Modification* (*ICD-9-CM*) codes.

**Main Outcomes and Measures:**

Dementia classified according to *ICD-9-CM* codes.

**Results:**

Of a total of 16 270 participants (9286 [57.1%] men; mean [SD] age, 65.2 [11.1] years), 1868 developed dementia. The dementia incidence rate in the HL group was higher than that in the non-HL group (19.38 [95% CI, 18.25-20.57] per 1000 person-years vs 13.98 [95% CI, 13.01-15.00] per 1000 person-years) during the follow-up period. In the fully adjusted multivariate Cox proportional hazards regression model applied for risk analysis, patients with HL had a significant risk of dementia (hazard ratio [HR], 1.17; 95% CI, 1.07-1.29; false discovery rate [FDR] *P* = .003). Subgroup analysis revealed that, among 3 age groups (45-64, 65-74, and ≥75 years), the group aged 45 to 64 years was associated with a risk of dementia (HR, 2.21 [95% CI, 1.57-3.12]; FDR *P* < .001). In sensitivity analysis, the presence of HL among those aged 45 to 64 years (HR, 1.40; 95% CI, 1.12-1.75; FDR *P* = .01) was associated with a risk of dementia.

**Conclusions and Relevance:**

In this study, hearing loss was positively associated with a risk of dementia, especially in patients aged 45 to 64 years. Hearing protection, screening, and treatment may be used as strategies for mitigating this potential risk factor.

## Key Points

**Question:**

What is the association of hearing loss with future incident dementia in the general population of Taiwan?

**Findings:**

In this population-based matched cohort study of 16 270 participants, hearing loss was positively associated with incident dementia, especially in patients aged 45 to 64 years.

**Meaning:**

Hearing loss is associated with a higher risk of dementia, and findings suggest that hearing protection, screening, and treatment may be used as strategies to mitigate this potential risk factor.

## Teaser

This population-based cohort study investigates the association of hearing loss with incident dementia among Taiwanese adults in the general population.

## Introduction

The prevalence of dementia has increased,^1^ with rapid increases in the elderly population. Dementia is associated with a higher risk of mortality, higher health care costs, and disability. Current treatment strategies only ameliorate symptoms and do not change the disease course. Identification of patients at risk of dementia is critical for preventing an impending dementia epidemic. Hearing loss (HL) has recently been recognized as a risk factor for dementia. The risk of HL increases with age and is associated with lower scores on tests of memory and a higher risk of incident all-cause dementia.^2,3^ Hearing loss includes peripheral hearing impairment and central auditory dysfunction; both are associated with accelerated cognitive decline, cognitive impairment, and incident dementia.^2,4,5,6^ Evidence suggests that even mild levels of HL increase the long-term risks of cognitive decline and dementia.^2,4,7,8,9^ Hearing loss in older adults may play a role in social isolation, depression, disability, lower quality of life, and risk of dementia^10^; however, HL is a possible biomarker of and modifiable risk factor for cognitive decline, cognitive impairment, and dementia.^11^ A meta-analysis^12^ stated that 65% of the risk factors for dementia are hereditary or genetic, which means they are not modifiable; however, 35% of the factors can be modified. Moreover, the adjusted population-attributable fraction of HL was calculated to be 9.1%; HL had the highest population-attributable fraction compared with other identified risk factors such as hypertension, obesity, depression, types 1 and 2 diabetes, and smoking. Thus, this longitudinal cohort study aimed to examine the association between HL and dementia.

## Methods

The institutional review board of National Taiwan Normal University approved this study. Written informed consent from the study patients was waived by the institutional review board because data were collected from the National Health Insurance Research Database (NHIRD), which consists of deidentified secondary data released for research purposes. This study followed the Strengthening the Reporting of Observational Studies in Epidemiology (STROBE) reporting guideline.

### Data Sources

This cohort study was conducted using data from the NHIRD.^13^ From this database, we collected data on outpatient care, inpatient care, ambulatory care, dental services, and prior medical conditions from January 1, 1997, through December 31, 2013. The National Health Insurance (NHI) program of Taiwan was established in 1995; it is a single-payer insurance system operated by the government that ensures the health of the entire nation and prevents social problems caused by poverty and disease. As of 2014, 99.9% of Taiwan’s population was enrolled in the NHI program.

### Study Design and Population

We conducted a population-based retrospective matched cohort study. Codes from *International Classification of Diseases, Ninth Revision, Clinical Modification* (*ICD-9-CM*) were used for disease diagnosis. Hearing loss was defined according to the *ICD-9-CM* codes 389 and A241 and included conductive, sensorineural, and mixed conductive, sensorineural, and unspecified HL. All medical claims between 1997 and 2013 containing these codes were obtained from the NHIRD for further analysis. Hearing loss with a duration of more than 1 year was considered, and at least 2 outpatient visits or any inpatient diagnosis was required. Patients newly diagnosed with HL from January 1, 2000, through December 31, 2011, were included in the exposed group (HL group); persons who were diagnosed with HL from 1997 through 1999 were excluded. Thus, 8135 patients newly diagnosed with HL without a dementia diagnosis before the index date were included. These exposed patients were compared with 8135 sex-, age-, residence-, and insurance premium–matched unexposed individuals (non-HL group) by exact matching. The index date was calculated by adding 365 days to the HL diagnosis date, and this date was assigned to the respective matched unexposed individuals, who were NHI enrollees without any HL or dementia diagnosis before the index date. Dementia (*ICD-9-CM* codes 290, 294.1, 331.0, 331.82, and A210) constituted the study outcome (eFigure 1 in the Supplement). Dementia was considered as an inpatient diagnosis or a diagnosis for outpatient services from the HL index date (HL diagnosis date plus 365 days).

### Potential Confounders

To better understand the association between HL and dementia, several potential confounders were included in this study. We defined potential confounders based on medical claims data from the NHIRD from January 1, 1997, to the index date. Demographic data included age, sex, residence, and insurance premium. The following general physical health data related to dementia were also collected: coronary artery disease (*ICD-9-CM* codes 410-415), cerebrovascular disease (*ICD-9-CM* codes 362.34 and 430-438), chronic obstructive pulmonary disease (*ICD-9-CM* codes 491, 492, 494, and 496), types 1 and 2 diabetes (*ICD-9-CM* codes 250 and 251), chronic kidney disease (*ICD-9-CM* codes 580-589), hypertension (*ICD-9-CM* codes 401-405), hyperlipidemia (*ICD-9-CM* code 272), anxiety (*ICD-9-CM* codes 300.0, 300.01, 300.02, 300.2, 300.21, 300.23, and 300.3), depression (*ICD-9-CM* codes 311, 296.2, 296.3, 296.82, 293.83, and 300.4), alcohol-related illness (*ICD-9-CM* codes 291, 303, 305.0, 571.0, 571.1, 571.2, 571.3, 790.3, A215, and V11.3), head injury (*ICD-9-CM* codes 800-804, 850-852, 853.1, 853.2, 854.0, 854.1, A290-291, and A470), rheumatoid arthritis (*ICD-9-CM* code 714), and asthma (*ICD-9-CM* code 493). The comorbidities included were required to be more than 1 year in duration and to have at least 2 outpatient visits or any inpatient diagnosis. Finally, the hospitalization length was included.

### Statistical Analysis

Data were analyzed from January 1, 2000, to December 31, 2013. The first dementia diagnosis of each person within the study period was used to calculate the risk of dementia during specific time intervals. The risk of dementia during the follow-up period was calculated using survival analysis. Follow-up was conducted from the index date to withdrawal from the NHI program or December 31, 2013, whichever occurred first. This study included incident dementia diagnosed from the index date (HL diagnosis date plus 365 days) to December 31, 2013. Multivariate Cox proportional hazards regression models adjusted for covariates were used to determine the associations of HL with dementia. The false discovery rate (FDR) and hazard ratios (HRs) with 95% CIs were calculated. False discovery–adjusted *P* values were used to avoid the inflation of false-positive findings. The model was applied for analyzing the entire study population. Subsequently, subgroups were formed according to sex and age, and the model was tested on these subgroups. Data analysis was performed with 2-sided tests using SAS, version 9.4 (SAS Institute Inc). *P* < .05 indicated significance.

### Sensitivity Analyses

To validate the robustness of the main study findings, we performed sensitivity analyses. Through subgroup analysis, the association of HL subgroups with dementia risk was evaluated. Furthermore, we used propensity score matching to control for sampling bias. Propensity scores represent patients’ probability of dementia incidence, and the scores were determined using a multivariate logistic regression model. The potential confounders used to calculate propensity scores included sex, age, residence, insurance premium, coronary artery disease, cerebrovascular disease, chronic obstructive pulmonary disease, diabetes, chronic kidney disease, severe liver disease, hyperlipidemia, anxiety, depression, alcohol-related illness, head injury, rheumatoid arthritis, asthma, and hospitalization length. The index year was defined as the year of the first HL diagnosis, and the index year was randomly selected for patients without HL. Using the estimated propensity scores, the HL and non-HL groups were matched at a ratio of 1:1 as closely as possible within each matched index year. Thus, the index year was the same between patients with HL and propensity score–matched individuals without HL. For balance diagnostics, the standardized difference was used to compare the mean of the binary variable between the HL and non-HL groups in propensity score–matched and nonmatched samples. A standardized difference of less than 0.1 indicated a negligible difference in the prevalence of a covariate between the HL and non-HL groups.^14^

## Results

### Population Characteristics

A total of 16 270 participants were included, of whom 9286 (57.1%) were men and 6984 (42.9%) were women (mean [SD] age, 65.2 [11.1] years). The exposed and unexposed groups included, respectively, 8135 patients with newly diagnosed HL (aged ≥45 years) and 8135 sex-, age-, residence-, and insurance premium–matched individuals without HL. Descriptions and a comparison of the groups’ characteristics are presented in Table 1 and eFigure 2 in the Supplement. Of patients with HL, 4643 (57.1%) were men, 3492 (42.9%) were women, and mean (SD) age was 65.2 (11.2) years. Urban residents constituted 5748 (70.7%), and 5179 (63.7%) were insurance premium dependent, had a low income, or had an insurance premium of less than NT$20 000. Coronary artery disease (2098 [25.8%] vs 1498 [18.4%]), cerebrovascular disease (1281 [15.7%] vs 909 [11.2%]), chronic obstructive pulmonary disease (1983 [24.4%] vs 1338 [16.4%]), diabetes (1801 [22.1%] vs 1460 [17.9%]), chronic kidney disease (714 [8.8%] vs 517 [6.4%]), hypertension (3955 [48.6%] vs 3482 [42.8%]), hyperlipidemia (2546 [31.3%] vs 1897 [23.3%]), anxiety (1762 [21.7%] vs 974 [12.0%]), depression (547 [6.7%] vs 282 [3.5%]), head injury (565 [6.9%] vs 378 [4.6%]), rheumatoid arthritis (199 [2.4%] vs 116 [1.4%]), and asthma (909 [11.2%] vs 719 [8.8%]) were more commonly observed in patients with HL than in the non-HL group (*P* < .001). The mean number of hospital admission days was higher in the HL group than in the non-HL group (2.78 [95% CI, 2.52-3.05] vs 1.83 [95% CI, 1.55-2.09] days; 2-tailed *t* test, *P* < .001).

**Table 1.** Characteristics of Enrollees With HL and Their Matched Controls in Taiwan

Characteristic	HL Group	Non-HL Group^a^	*P* Value^b^
Sex, No. (%)			
Male	4643 (57.1)	4643 (57.1)	Exact match
Female	3492 (42.9)	3492 (42.9)	
Age, mean (SD), y	65.2 (11.2)	65.1 (11.1)	Exact match
Age group, No. (%), y			
45-64	2914 (35.8)	2914 (35.8)	Exact match
65-74	3424 (42.1)	3424 (42.1)	
≥75	1797 (22.1)	1797 (22.1)	
Insurance premium, No. (%), NT$^c^			
Fixed premium, dependent, low income	2844 (35.0)	2844 (35.0)	Exact match
<20 000	2335 (28.7)	2335 (28.7)	
≥20 000	2956 (36.3)	2956 (36.3)	
Residence, No. (%)			
Rural	2387 (29.3)	2387 (29.3)	Exact match
Urban	5748 (70.7)	5748 (70.7)	
Coronary artery disease, No. (%)			
No	6037 (74.2)	6637 (81.6)	<.001
Yes	2098 (25.8)	1498 (18.4)	
Cerebrovascular disease, No. (%)			
No	6854 (84.3)	7226 (88.8)	<.001
Yes	1281 (15.7)	909 (11.2)	
Chronic obstructive pulmonary disease, No. (%)			
No	6152 (75.6)	6797 (83.6)	<.001
Yes	1983 (24.4)	1338 (16.4)	
Diabetes, No. (%)			
No	6334 (77.9)	6675 (82.1)	<.001
Yes	1801 (22.1)	1460 (17.9)	
Chronic kidney disease, No. (%)			
No	7421 (91.2)	7618 (93.6)	<.001
Yes	714 (8.8)	517 (6.4)	
Hypertension, No. (%)			
No	4180 (51.4)	4653 (57.2)	<.001
Yes	3955 (48.6)	3482 (42.8)	
Hyperlipidemia, No. (%)			
No	5589 (68.7)	6238 (76.7)	<.001
Yes	2546 (31.3)	1897 (23.3)	
Anxiety, No. (%)			
No	6373 (78.3)	7161 (88.0)	<.001
Yes	1762 (21.7)	974 (12.0)	
Depression, No. (%)			
No	7588 (93.3)	7853 (96.5)	<.001
Yes	547 (6.7)	282 (3.5)	
Alcohol-related illness, No. (%)			
No	7987 (98.2)	8024 (98.6)	.02
Yes	148 (1.8)	111 (1.4)	
Head injury, No. (%)			
No	7570 (93.1)	7757 (95.4)	<.001
Yes	565 (6.9)	378 (4.6)	
Rheumatoid arthritis, No. (%)			
No	7936 (97.6)	8019 (98.6)	<.001
Yes	199 (2.4)	116 (1.4)	
Asthma, No. (%)			
No	7226 (88.8)	7416 (91.2)	<.001
Yes	909 (11.2)	719 (8.8)	
Dementia, No. (%)			
No	7041 (86.6)	7361 (90.5)	<.001
Yes	1094 (13.4)	774 (9.5)	
Length of hospital admission, mean (95% CI), d^d^	2.78 (2.52-3.05)	1.83 (1.55-2.09)	<.001

Abbreviation: HL, hearing loss.

^a^ Includes sex-, age-, residence-, and insurance premium–matched controls.

^b^ Calculated using the χ^2^ or the 2-tailed *t* test. Testing was not necessary for exact matching samples.

^c^ US$1 = NT$31.2 in 2019.

^d^ Indicates admission to a hospital 1 year before the diagnosis of HL.

### Dementia Incidence

Of a total of 16 270 participants, 1868 developed dementia during the follow-up period. Among them, 1094 (58.6%) were from the HL group and 774 (41.4%) were from the non-HL group, as shown in the Figure. The mean (SD) follow-up interval for all patients was 6.87 (3.36) years, and the total follow-up intervals were 56 432.01 and 55 355.47 person-years for the HL and non-HL groups, respectively (Table 2). The dementia incidence rates were 19.38 (95% CI, 18.25-20.57) per 1000 person-years in the HL group and 13.98 (95% CI, 13.01-15.00) per 1000 person-years in the non-HL group. Kaplan-Meier analysis of the cumulative incidence of dementia revealed that dementia incidence rates were significantly higher in the HL group than in the non-HL group (*P* < .001 according to the log-rank test).

**Figure.**
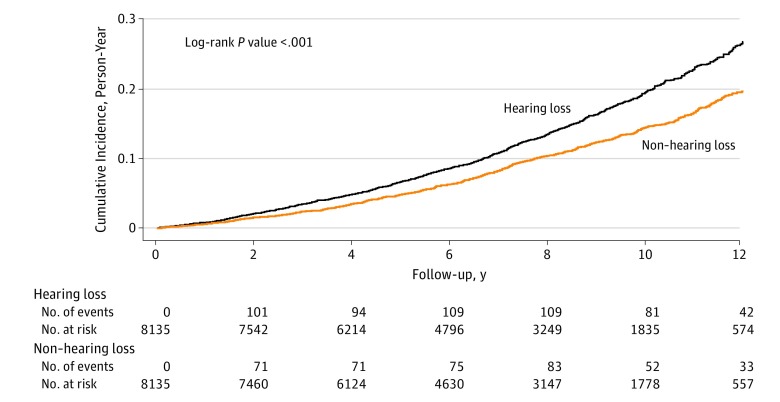
Cumulative Incidence of Dementia by Study Group The 8135 patients with hearing loss (HL group) were matched by sex, age, residence, and insurance premium with 8135 individuals without hearing loss (non-HL group).

**Table 2.** Incidence Rate of Dementia in Taiwan

Characteristic	No. of Events	Person-Years	Incidence Rate (95% CI)^a^	Multivariate Cox Proportional Hazards Regression Analysis				
				Unadjusted		Adjusted^b^		
				HR (95% CI)	*P* Value	HR (95% CI)	*P* Value	FDR *P* Value
Hearing loss								
No	774	55 355.47	13.98 (13.01-15.00)	1 [Reference]	<.001	1 [Reference]	.001	.003
Yes	1094	56 432.01	19.38 (18.25-20.57)	1.38 (1.26-1.51)		1.17 (1.07-1.29)		
Sex								
Male	1114	63 589.56	17.52 (16.50-18.58)	1.12 (1.02-1.23)	.02	0.90 (0.81-0.99)	.04	.06
Female	754	48 197.93	15.64 (14.55-16.80)	1 [Reference]		1 [Reference]		
Age^c^	NA	NA	NA	2.66 (2.53-2.79)	<.001	2.55 (2.42-2.69)	<.001	<.001
Insurance premium, NT$^d^								
Fixed premium, dependent, low income	644	39 232.14	16.41 (15.17-17.73)	1 [Reference]	NA	1 [Reference]	NA	NA
<20 000	749	31 658.65	23.66 (21.99-25.42)	1.46 (1.31-1.62)	<.001	0.97 (0.87-1.09)	.72	.70
≥20 000	475	40 896.70	11.61 (10.59-14.43)	0.71 (0.63-0.80)	<.001	0.71 (0.62-0.81)	<.001	<.001
Residence								
Rural	557	32 953.09	16.90 (15.52-18.37)	1 [Reference]	.77	1 [Reference]	.03	.05
Urban	1311	78 834.39	16.62 (15.74-17.55)	0.99 (0.89-1.09)		0.88 (0.78-0.99)		
Coronary artery disease								
No	1275	90 304.39	14.11 (13.35-14.92)	1 [Reference]	<.001	1 [Reference]	.001	.01
Yes	593	21 483.09	27.60 (25.43-29.92)	2.16 (1.96-2.38)		1.16 (1.04-1.29)		
Cerebrovascular disease								
No	1441	99 009.76	14.55 (13.81-15.32)	1 [Reference]	<.001	1 [Reference]	<.001	<.001
Yes	427	12 777.72	33.41 (30.32-36.74)	2.51 (2.25-2.79)		1.36 (1.21-1.52)		
Chronic obstructive pulmonary disease								
No	1346	91384.42	14.73 (13.95-15.54)	1 [Reference]	<.001	1 [Reference]	.62	.70
Yes	522	20 403.06	25.58 (23.44-27.88)	1.88 (1.70-2.08)		0.97 (0.87-1.09)		
Diabetes								
No	1388	92 053.95	15.07 (14.30-15.89)	1 [Reference]	<.001	1 [Reference]	<.001	<.001
Yes	480	19 733.53	24.32 (22.20-26.60)	1.76 (1.58-1.95)		1.29 (1.15-1.44)		
Chronic kidney disease								
No	1675	104 670.24	16.00 (15.25-16.79)	1 [Reference]	<.001	1 [Reference]	.67	.70
Yes	193	7117.25	27.12 (23.43-31.22)	1.86 (1.60-2.16)		1.03 (0.89-1.21)		
Hypertension								
No	788	65 581.22	12.02 (11.19-12.88)	1 [Reference]	<.001	1 [Reference]	.09	.13
Yes	1080	46 206.27	23.37 (22.00-24.81)	2.16 (1.97-2.37)		1.09 (0.99-1.21)		
Hyperlipidemia								
No	1357	84 850.46	15.99 (15.15-16.87)	1 [Reference]	<.001	1 [Reference]	.63	.70
Yes	511	26 937.03	18.97 (17.36-20.69)	1.31 (1.18-1.45)		0.97 (0.87-1.09)		
Anxiety								
No	1473	95 029.25	15.50 (14.72-16.31)	1 [Reference]	<.001	1 [Reference]	<.001	<.001
Yes	395	16 758.24	23.57 (21.30-26.01)	1.65 (1.47-1.84)		1.28 (1.13-1.45)		
Depression								
No	1715	106 903.03	16.04 (15.29-16.82)	1 [Reference]	<.001	1 [Reference]	<.001	<.001
Yes	153	4884.45	31.32 (26.56-36.70)	2.12 (1.80-2.50)		1.50 (1.25-1.79)		
Alcohol-related illness								
No	1833	110 306.72	16.61 (15.87-17.40)	1 [Reference]	.01	1 [Reference]	.002	.004
Yes	35	1480.76	23.64 (16.46-32.87)	1.55 (1.11-2.17)		1.71 (1.22-2.41)		
Head injury								
No	1717	106 006.29	16.20 (15.44-16.98)	1 [Reference]	<.001	1 [Reference]	.001	.003
Yes	151	5781.19	26.12 (22.12-30.63)	1.70 (1.44-2.01)		1.33 (1.12-1.57)		
Rheumatoid arthritis								
No	1819	109 880.12	16.55 (15.80-17.33)	1 [Reference]	<.001	1 [Reference]	.07	.10
Yes	49	1907.37	25.69 (19.01-33.96)	1.69 (1.27-2.25)		1.31 (0.98-1.74)		
Asthma								
No	1648	102 001.40	16.16 (15.39-16.96)	1 [Reference]	<.001	1 [Reference]	>.99	>.99
Yes	220	9786.08	22.48 (19.61-25.66)	1.51 (1.31-1.74)		1.00 (0.86-1.16)		
Length of hospital stay^e^	NA	NA	NA	1.58 (1.30-1.91)	<.001	1.26 (0.90-1.77)	.18	.25

Abbreviations: FDR, false discovery rate; HR, hazard ratio; NA, not applicable.

^a^ Indicates per 1000 person-years.

^b^ Adjusted for sex, age, residence, insurance premium, coronary artery disease, chronic obstructive pulmonary disease, cerebrovascular disease, diabetes, chronic kidney disease, hypertension, hyperlipidemia, anxiety, depression, alcohol-related illness, head injury, rheumatoid arthritis, asthma, and length of hospital stay.

^c^ Indicates mean age at date of diagnosis of hearing loss divided by 10.

^d^ US$1 = NT$31.2 in 2019.

^e^ Indicates number of admission days divided by 100.

### HL and the Risk of Dementia

As shown in Table 2, a high rate of dementia incidence (>20.00 per 1000 person-years) was found in patients with cerebrovascular disease, coronary artery disease, chronic obstructive pulmonary disease, diabetes, chronic kidney disease, hypertension, anxiety, depression, alcohol-related illnesses, head injury, rheumatoid arthritis, and asthma compared with patients without these conditions. In the fully adjusted multivariate Cox proportional hazards regression model for risk analysis, HL was positively associated with dementia (HR, 1.17; 95% CI, 1.07-1.29; FDR *P* = .003). Advanced age (HR, 2.55; 95% CI, 2.42-2.69; FDR *P* < .001) was significantly and positively associated with dementia; however, a high insurance premium (HR, 0.71; 95% CI, 0.62-0.81; FDR *P* < .001) was significantly and negatively associated with dementia. Moreover, cerebrovascular disease (HR, 1.36; 95% CI, 1.21-1.52), diabetes (HR, 1.29; 95% CI, 1.15-1.44), anxiety (HR, 1.28; 95% CI, 1.13-1.45), depression (HR, 1.50; 95% CI, 1.25-1.79), alcohol-related illnesses (HR, 1.71; 95% CI, 1.22-2.41), and head injury (HR, 1.33; 95% CI, 1.12-1.57) were significantly and positively associated with dementia (FDR *P* < .01 for all) according to the fully adjusted multivariate Cox proportional hazards regression model for risk analysis. Subgroup analysis by sex and age groups (eTable in the Supplement) indicated that male sex was significantly and positively associated with dementia (HR, 1.20; 95% CI, 1.06-1.36; FDR *P* = .008) and that the group aged 45 to 64 years was positively associated with dementia (HR, 2.21; 95% CI, 1.57-3.12; FDR *P* < .001).

### Sensitivity and Subgroup Analyses

The distributions of potential confounders were similar between the HL and non-HL groups after propensity score matching (standardized difference, <0.1) (Table 3). The results obtained from sensitivity analysis (Table 4) were applied to the fully adjusted Cox proportional hazards regression model, which revealed a nonsignificant association between HL and dementia risk (HR, 1.11; 95% CI, 1.01-1.22; FDR *P* = .051). Advanced age (HR, 2.49; 95% CI, 2.36-2.62), cerebrovascular disease (HR, 1.26; 95% CI, 1.12-1.41), diabetes (HR, 1.32; 95% CI, 1.19-1.47), anxiety (HR, 1.28; 95% CI, 1.14-1.44), depression (HR, 1.62; 95% CI, 1.37-1.91), and head injury (HR, 1.27; 95% CI, 1.08-1.49) were significantly and positively associated with dementia (FDR *P* < .01 for all), and the highest insurance premium was significantly and negatively associated with dementia (HR, 0.69; 95% CI, 0.61-0.79; FDR *P* < .001). These findings were similar to the results of our main analysis. Subgroup analyses revealed that, among the 3 age groups (eTable in the Supplement), the group aged 45 to 64 years was associated with a significant risk of dementia (HR, 1.40; 95% CI, 1.12-1.75; FDR *P* = .01). Male sex was not associated with a significant risk of dementia. Segment-adjusted analysis showed that HL was not significantly associated with dementia 1 to 4 years after the index date (HR, 1.17; 95% CI, 1.02-1.35; FDR *P* = .06). The association was significant 5 to 12 years after the index date (HR, 1.17; 95% CI, 1.04-1.33; FDR *P* = .04) in exact matching analysis; the association between HL and dementia 1 to 4 years after the index date (HR, 1.07; 95% CI, 0.94-1.23; FDR *P* = .39) and the association 5 to 12 years after the index date (HR, 1.15; 95% CI, 1.01-1.30; FDR *P* = .07) in propensity score matching analysis were not significant.

**Table 3.** Demographic and Clinical Characteristics of the Study Population Based on HL Diagnosis and Propensity Score Matching

Characteristic	Before Matching^a^			After Matching^a^		
	No. (%)		Standardized Difference	No. (%)		Standardized Difference
	HL Group	Non-HL Group		HL Group	Non-HL Group	
Sex						
Male	4643 (57.1)	453 663 (49.4)	0.15	4643 (57.1)	4461 (54.8)	0.04
Female	3492 (42.9)	464 884 (50.6)	0.15	3492 (42.9)	3674 (45.2)	0.04
Age group, y						
45-64	3960 (48.7)	846 742 (92.2)	1.08	3960 (48.7)	3772 (46.4)	0.05
65-74	2378 (29.2)	45 997 (5.0)	0.68	2378 (29.2)	2335 (28.7)	0.01
≥75	1797 (22.1)	25 808 (2.8)	0.61	1797 (22.1)	2028 (24.9)	0.07
Insurance premium, NT$^b^						
Fixed premium, dependent, low income	2844 (35.0)	293 091 (31.9)	0.06	2844 (35.0)	3136 (38.5)	0.07
<20 000	2335 (28.7)	196 648 (21.4)	0.17	2335 (28.7)	2201 (27.1)	0.04
≥20 000	2956 (36.3)	428 808 (46.7)	0.21	2956 (36.3)	2798 (34.4)	0.04
Residence						
Rural	2387 (29.3)	190 897 (20.8)	0.20	2387 (29.3)	2385 (29.3)	0.0004
Urban	5748 (70.7)	727 650 (79.2)	0.20	5748 (70.7)	5750 (70.7)	0.0004
Index year						
2000	890 (10.9)	83 336 (9.1)	0.06	890 (10.9)	890 (10.94)	Exact match
2001	853 (10.5)	81 582 (8.9)	0.05	853 (10.5)	853 (10.5)	Exact match
2002	812 (10.0)	80 823 (8.8)	0.04	812 (10.0)	812 (10.0)	Exact match
2003	754 (9.3)	80 773 (8.8)	0.02	754 (9.3)	754 (9.3)	Exact match
2004	815 (10.0)	79 627 (8.7)	0.05	815 (10.0)	815 (10.0)	Exact match
2005	754 (9.3)	77 122 (8.4)	0.03	754 (9.3)	754 (9.3)	Exact match
2006	687 (8.4)	75 121 (8.2)	0.01	687 (8.4)	687 (8.4)	Exact match
2007	628 (7.7)	74 109 (8.1)	0.01	628 (7.7)	628 (7.7)	Exact match
2008	603 (7.4)	72 967 (7.9)	0.02	603 (7.4)	603 (7.4)	Exact match
2009	558 (6.9)	71 568 (7.8)	0.04	558 (6.9)	558 (6.9)	Exact match
2010	459 (5.6)	70 843 (7.7)	0.08	459 (5.6)	459 (5.6)	Exact match
2011	322 (4.0)	69 891 (7.6)	0.16	322 (4.0)	322 (4.0)	Exact match
Coronary artery disease						
No	6037 (74.2)	878 706 (95.7)	0.63	6041 (74.3)	6252 (76.9)	0.06
Yes	2098 (25.8)	39 841 (4.3)	0.63	2094 (25.7)	1883 (23.1)	0.06
Cerebrovascular disease						
No	6854 (84.3)	897 336 (97.7)	0.48	6854 (84.3)	7092 (87.2)	0.08
Yes	1281 (15.7)	21 211 (2.3)	0.48	1281 (15.7)	1043 (12.8)	0.08
Chronic obstructive pulmonary disease						
No	6152 (75.6)	879 576 (95.8)	0.60	6152 (75.6)	6418 (78.9)	0.08
Yes	1983 (24.4)	38 971 (4.2)	0.60	1983 (24.4)	1717 (21.1)	0.08
Diabetes						
No	6334 (77.9)	869 840 (94.7)	0.50	6334 (77.9)	6457 (79.4)	0.04
Yes	1801 (22.1)	48 707 (5.3)	0.50	1801 (22.1)	1678 (20.6)	0.04
Chronic kidney disease						
No	7421 (91.2)	901 083 (98.1)	0.31	7421 (91.2)	7544 (92.7)	0.06
Yes	714 (8.8)	17 464 (1.9)	0.31	714 (8.8)	591 (7.3)	0.06
Hypertension						
No	4180 (51.4)	812 283 (88.4)	0.88	4180 (51.4)	4214 (51.8)	0.01
Yes	3955 (48.6)	106 264 (11.6)	0.88	3955 (48.6)	3921 (48.2)	0.01
Hyperlipidemia						
No	5589 (68.7)	841 754 (91.6)	0.60	5589 (68.7)	5722 (70.3)	0.04
Yes	2546 (31.3)	76 793 (8.4)	0.60	2546 (31.3)	2413 (29.7)	0.04
Anxiety						
No	6373 (78.3)	867 351 (94.4)	0.48	6373 (78.3)	6559 (80.6)	0.06
Yes	1762 (21.7)	51 196 (5.6)	0.48	1762 (21.7)	1576 (19.4)	0.06
Depression						
No	7588 (93.3)	900 005 (98.0)	0.23	7588 (93.3)	7705 (94.7)	0.06
Yes	547 (6.7)	18 542 (2.0)	0.23	547 (6.7)	430 (5.3)	0.06
Alcohol-related illness						
No	7987 (98.2)	911 467 (99.2)	0.09	7987 (98.2)	8001 (98.4)	0.01
Yes	148 (1.8)	7080 (0.8)	0.09	148 (1.8)	134 (1.6)	0.01
Head injury						
No	7570 (93.1)	888 807 (96.8)	0.17	7570 (93.1)	7661 (94.2)	0.05
Yes	565 (6.9)	29 740 (3.2)	0.17	565 (6.9)	474 (5.8)	0.05
Rheumatoid arthritis						
No	7936 (97.6)	913 285 (99.4)	0.15	7936 (97.6)	7975 (98.0)	0.03
Yes	199 (2.4)	5262 (0.6)	0.15	199 (2.4)	160 (2.0)	0.03
Asthma						
No	7226 (88.8)	866 717 (94.4)	0.20	7226 (88.8)	7329 (90.1)	0.04
Yes	909 (11.2)	51 830 (5.6)	0.20	909 (11.2)	806 (9.9)	0.04
Admission^c^						
No	6132 (75.4)	856 284 (93.2)	0.42	6132 (75.4)	6356 (78.1)	0.06
Yes	2003 (24.6)	62 263 (6.8)	0.42	2003 (24.6)	1779 (21.9)	0.06

Abbreviation: HL, hearing loss.

^a^ Propensity score matching was performed for sex, age, residence, insurance premium, coronary artery disease, chronic obstructive pulmonary disease, cerebrovascular disease, diabetes, chronic kidney disease, hypertension, hyperlipidemia, anxiety, depression, alcohol-related illness, head injury, rheumatoid arthritis, asthma, times of hospital admission, and index year.

^b^ US$1 = NT$31.2 in 2019.

^c^ Indicates admission to a hospital 1 year before a diagnosis of HL.

**Table 4.** Propensity Score Matching to Reveal the Association Between Clinical Characteristics and Dementia Risk in Taiwan

Characteristic	All Participants (n = 16 270)				
	Unadjusted Analysis		Adjusted Analysis^a^		
	HR (95% CI)	*P* Value	HR (95% CI)	*P* Value	FDR *P* Value
Hearing loss					
No	1 [Reference]	<.001	1 [Reference]	.02	.051
Yes	1.18 (1.08-1.29)		1.11 (1.01-1.22)		
Sex					
Male	1.10 (1.00-1.20)	.04	1.02 (0.92-1.12)	.71	.79
Female	1 [Reference]		1 [Reference]		
Age^b^	2.55 (2.43-2.67)	<.001	2.49 (2.36-2.62)	<.001	<.001
Insurance premium, NT$^c^					
Fixed premium, dependent, low income	1 [Reference]	NA	1 [Reference]	NA	NA
<20 000	1.25 (1.12-1.38)	<.001	0.89 (0.80-0.99)	.06	.70
≥20 000	0.67 (0.60-0.75)	<.001	0.69 (0.61-0.79)	<.001	<.001
Residence					
Rural	1 [Reference]	.45	1 [Reference]	.08	.13
Urban	0.96 (0.87-1.06)		0.91 (0.81-1.01)		
Coronary artery disease					
No	1 [Reference]	<.001	1 [Reference]	.32	.38
Yes	1.99 (1.81-2.19)		1.05 (0.95-1.17)		
Cerebrovascular disease					
No	1 [Reference]	<.001	1 [Reference]	<.001	<.001
Yes	2.39 (2.15-2.66)		1.26 (1.12-1.41)		
Chronic obstructive pulmonary disease					
No	1 [Reference]	<.001	1 [Reference]	.87	.87
Yes	1.81 (1.64-1.99)		1.01 (0.91-1.12)		
Diabetes					
No	1 [Reference]	<.001	1 [Reference]	<.001	<.001
Yes	1.75 (1.59-1.94)		1.32 (1.19-1.47)		
Chronic kidney disease					
No	1 [Reference]	<.001	1 [Reference]	.87	.87
Yes	1.89 (1.63-2.19)		1.01 (0.87-1.18)		
Hypertension					
No	1 [Reference]	<.001	1 [Reference]	.03	.05
Yes	2.17 (1.98-2.38)		1.12 (1.01-1.24)		
Hyperlipidemia					
No	1 [Reference]	<.001	1 [Reference]	.09	.13
Yes	1.32 (1.20-1.45)		1.10 (0.99-1.22)		
Anxiety					
No	1 [Reference]	<.001	1 [Reference]	<.001	<.001
Yes	1.50 (1.35-1.67)		1.28 (1.14-1.44)		
Depression					
No	1 [Reference]	<.001	1 [Reference]	<.001	<.001
Yes	2.06 (1.77-2.40)		1.62 (1.37-1.91)		
Alcohol-related illness					
No	1 [Reference]	.10	1 [Reference]	.09	.13
Yes	1.32 (0.94-1.84)		1.34 (0.96-1.88)		
Head injury					
No	1 [Reference]	<.001	1 [Reference]	.004	.01
Yes	1.55 (1.32-1.82)		1.27 (1.08-1.49)		
Rheumatoid arthritis					
No	1 [Reference]	.01	1 [Reference]	.27	.33
Yes	1.44 (1.08-1.91)		1.17 (0.88-1.56)		
Asthma					
No	1 [Reference]	<.001	1 [Reference]	.22	.28
Yes	1.65 (1.44-1.88)		1.09 (0.95-1.26)		
Length of hospital stay, d^d^	1.25 (1.14-1.37)	<.001	1.26 (1.11-1.42)	<.001	<.001

Abbreviations: FDR, false discovery rate; HR, hazard ratio; NA, not applicable.

^a^ Adjusted for sex, age, residence, insurance premium, coronary artery disease, chronic obstructive pulmonary disease, cerebrovascular disease, diabetes, chronic kidney disease, hypertension, hyperlipidemia, anxiety, depression, alcohol-related illnesses, head injury, rheumatoid arthritis, asthma, length of hospital stay, and outpatient visits.

^b^ Indicates mean age at date of diagnosis of hearing loss, divided by 10.

^c^ US$1 = NT$31.2 in 2019.

^d^ Indicates number of admission days divided by 100.

In the group aged 45 to 64 years, the interactions between head injury and HL (Wald χ^2^_1_ test, 2.19; *P* = .14) or between alcohol-related illness and HL (Wald χ^2^_1_ test, 0.08; *P* = .77) were not significant. In the group aged 65 to 74 years, the interaction between head injury and HL was significant (Wald χ^2^_1_ test, 4.48; *P* = .03), and the interaction between alcohol-related illness and HL was not significant (Wald χ^2^_1_ test, 0.98; *P* = .32). In the group aged 75 years or older, the interactions between head injury and HL (Wald χ^2^_1_ test, 1.29; *P* = .25) or between alcohol-related illness and HL (Wald χ^2^_1_ test, 0.86; *P* = .35) were not significant.

## Discussion

The findings of the present population-based cohort study indicate that HL is positively associated with the risk of dementia. These results were robust after adjustments for multiple confounders, and patients with HL had a significantly higher risk of dementia. Subgroup analyses revealed that, among the 3 age groups, the group aged 45 to 64 years was associated with a significant risk of dementia.

The main analysis results demonstrated that in the HL group, patients aged 45 to 64 years showed significantly stronger and more positive associations with dementia occurrence than patients aged 65 to 74 years or 75 years or older compared with their counterparts in the non-HL group. Patients with HL aged 45 to 64 years were characterized by a higher HR, whereas similar findings were not obtained for patients aged 65 to 74 years or 75 years or older. Previous studies^3,4^ have indicated that age-related HL, such as presbycusis and sensory HL,^2,7,9,15^ is associated with the incidence of dementia and even development of Alzheimer disease (AD). However, scant evidence suggests an increased risk of dementia or AD in middle-aged patients with HL, and this research topic has received little attention previously. Recently, Livingston et al^12^ observed that preventing HL in midlife may modify the 9.1% risk of incident dementia. A recent cohort study^16^ reported that midlife HL is associated with an increased rate of dementia at younger than 60 years, and the association between HL and cognition may be due to shared etiology or because HL causes cognitive reduction through impaired speech perception in younger individuals and increased use of executive function and working memory in older people, resulting in the constant effort. These findings support the association between midlife HL and dementia.

Several neural changes have been described in people with hearing impairment. Age-related HL may result in the acceleration of aging because the peripheral and central nervous systems can alter synapses and neural anatomy with aging.^17^ To compensate for decreased auditory input owing to HL, increased listening effort is required through the additional recruitment of frontal areas.^18^ An increase in cognitive load occurs in patients who are hard of hearing; consequently, cognitive processes such as memory and executive function are adversely affected.^19,20^ Hearing loss, defined as sensory deprivation, may alter the usual pattern of resource allocation in the brain, thereby affecting neural reserves and cognitive performance.^20,21^ Hearing loss leads to altered auditory processing, and research has shown that cross-modal neuroplasticity changes in the auditory cortex and altered visual and audiovisual information processing affect functional connectivity between the sensory cortices.^18,19,22^ Hence, early HL development may causally accelerate atrophy in the entire brain, which could lead to cognitive reserve depletion in the brain.^23^

Hearing loss is associated with difficulties in understanding speech, especially under adverse listening conditions,^24^ and patients with HL may experience long-term disturbances in life, such as difficulties in communication, social isolation, and bad mood, which further suggests that HL may be associated with dementia.^25^ Intervention studies have indicated that hearing aids are associated with higher cognitive performance, and this association is mediated through social isolation or depression. Higher levels of HL are associated with an increased risk of social isolation,^26,27^ and social isolation and depression have been found to exacerbate cognitive impairment.^28^

Hearing loss is a potential reversible risk factor for dementia and AD.^12,17,29^ Studies^30,31^ have demonstrated that hearing aids and cochlear implants impede cognitive decline. Hence, the early identification of HL in patients and successful hearing rehabilitation can mitigate the negative effects of HL.^12^ However, the ideal time to perform HL screening to reduce the risk of dementia remains unclear. The findings of the present study suggest that screening for HL should be performed when people are middle aged.

In the present study, 6 comorbidities were significantly associated with an increased risk of dementia (FDR *P* < .01) in fully adjusted analysis, namely, cerebrovascular disease, diabetes, anxiety, depression, alcohol-related illnesses, and head injury. This finding implies a strong association between these comorbidities and dementia risk. An increasing number of studies have reported that cerebrovascular disease, diabetes, anxiety, depression, and head injury are risk factors for dementia, and these conditions might interrupt cognitive reserve function (as discussed previously)^12^; however, alcohol-related illnesses did not show such an association. Chronic alcoholism adversely affects the brain, causing cognitive impairment.^32,33^ A study indicated that alcohol use–related disorders are associated with a significantly higher risk of dementia.^34^ Other studies have shown that frequent alcohol consumption is associated with an increased risk of dementia compared with infrequent alcohol consumption.^35^ However, a systematic review^36^ indicated that light to moderate alcohol consumption may protect against AD and dementia. This discrepancy may be due to the case definition. That study included patients with confirmed diagnoses related to alcoholism, and previous studies have included patients based on self-report drinking behavior questionnaires.

### Strengths and Limitations

This cohort study was based on nationwide representative data, and potential confounders such as social and demographic factors and the general health status were controlled for in the adjusted multivariate Cox proportional hazards regression models in the main analysis and through propensity score matching in the sensitivity analysis. However, the principal limitation with any study of this nature is that causality is difficult to infer, and we can only state that findings support a given hypothesis. In addition, the main limitation of studies based on the insurance database is the lack of precision in dementia diagnosis. Moreover, the database contains limited or no information on other potential confounders such as educational level, body mass index, physical activities, diet pattern, blood pressure, blood glucose levels, smoking, and use of hearing aids. Information on dementia subtype and HL severity is also lacking in the study database. Disease onset and diagnosis may differ according to the economic status and residence of patients because these variables affect access to neurologists. The comorbidity rate in patients with HL was higher than that in the non-HL group; patients with HL are possibly diagnosed with dementia earlier due to frequent outpatient visits. The HL group might have more opportunities for dementia diagnoses than the non-HL group. We used propensity score matching in sensitivity analysis to address this potential bias. Our segment analysis showed that the association of HL with dementia was significant at 5 to 12 years and not at 1 to 4 years after the index date of HL. Therefore, this bias was weak in our study. Despite the limitations of this study, the association between HL and dementia was consistent with many other investigations in the literature.^2,3,12^

## Conclusions

In this cohort study, HL was associated with a significant increase in the risk of dementia, especially in patients aged 45 to 64 years. This finding suggests that the implementation of early hearing protection, HL screening, and the use of hearing aids may help to mitigate this potential risk factor for dementia.

## Notes

Supplement. **eFigure 1.** Flowchart of Database Assembly and Cohort Study Cases Selection **eFigure 2.** Age Distribution in Non–Hearing Loss and Hearing Loss Groups **eTable.** Effect of Hearing Loss on Dementia in Taiwan by Sex and Age Groups, Cohort Study, 2000 to 2011
