# Supplementary material for: Association of Hearing Loss With Dementia
Source: JAMA Netw Open. 2019 Jul 31;2(7):e198112. doi: 10.1001/jamanetworkopen.2019.8112 (PMC6669778; doi:10.1001/jamanetworkopen.2019.8112)
Supplement: Supplement. — eFigure 1. Flowchart of Database Assembly and Cohort Study Cases Selection eFigure 2. Age Distribution in Non–Hearing Loss and Hearing Loss Groups eTable. Effect of Hearing Loss on Dementia in Taiwan by Sex and Age Groups, Cohort Study, 2000 to 2011 [file jamanetwopen-2-e198112-s001.pdf]

Supplementary Online Content

Liu C-M, Lee CT-C. Association of hearing loss with dementia. *JAMA Netw Open.* 2019;2(7):e198112. doi:10.1001/jamanetworkopen.2019.8112

eFigure 1. Flowchart of Database Assembly and Cohort Study Cases Selection
eFigure 2. Age Distribution in Non–Hearing Loss and Hearing Loss Groups
eTable. Effect of Hearing Loss on Dementia in Taiwan by Sex and Age Groups, Cohort Study, 2000 to 2011

This supplementary material has been provided by the authors to give readers additional information about their work.

© 2019 Liu C-M et al. *JAMA Network Open.*

eFigure 1. Flowchart of Database Assembly and Cohort Study Cases Selection

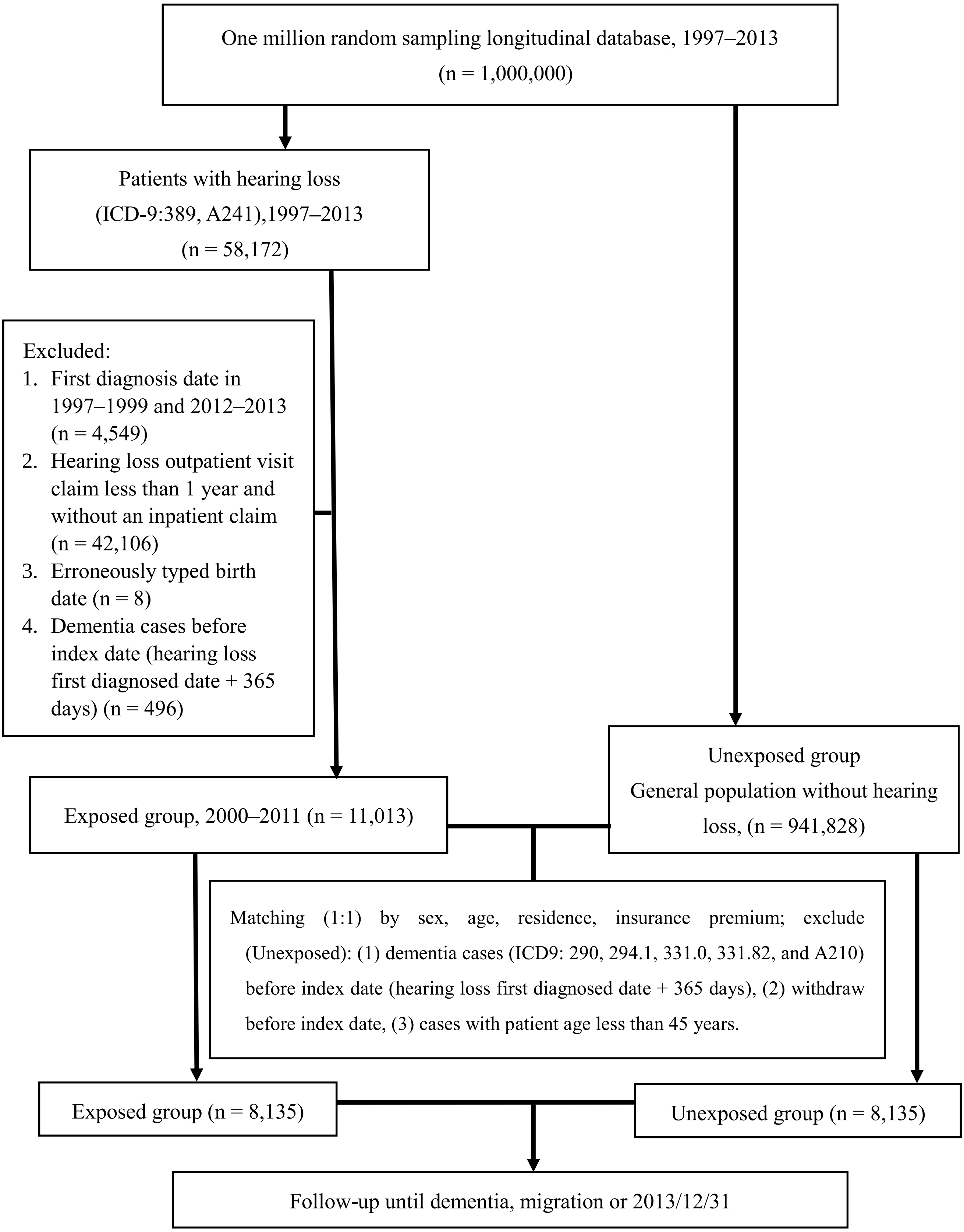

© 2019 Liu C-M et al. *JAMA Network Open.*

eFigure 2. Age Distribution in Non–Hearing Loss and Hearing Loss Groups

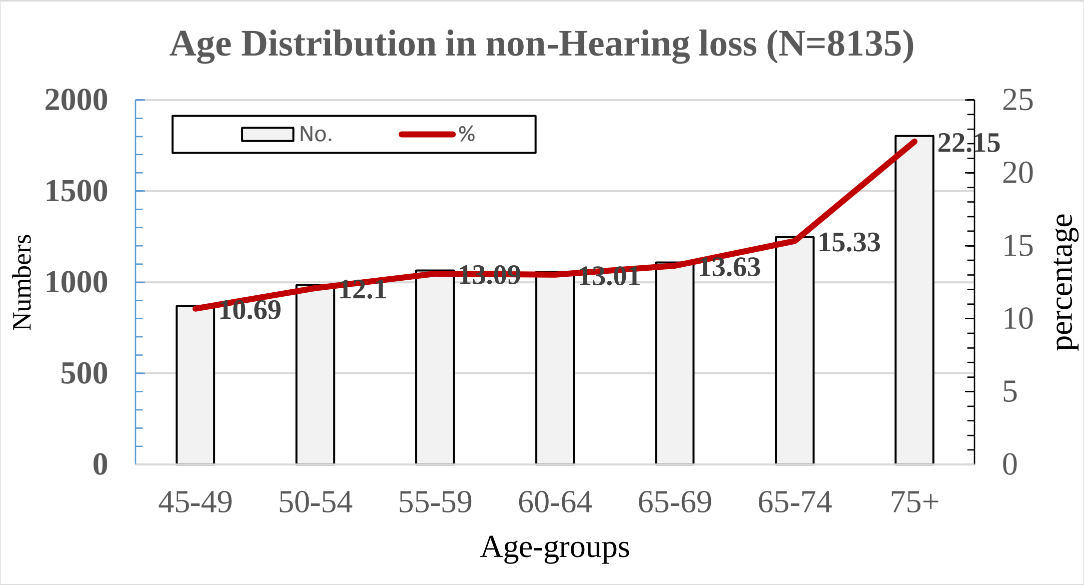

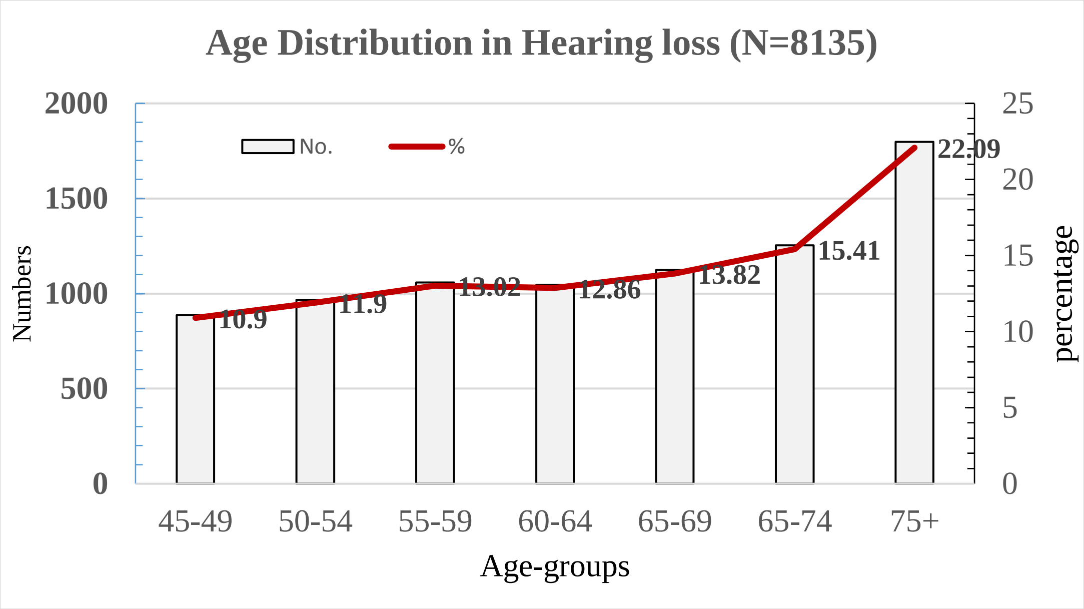

© 2019 Liu C-M et al. *JAMA Network Open.*

eTable. Effect of Hearing Loss on Dementia in Taiwan by Sex and Age Groups, Cohort Study, 2000 to 2011

Subgroup analysis	Exact matching					Propensity score matching		
	Incidence rate (95% CI) (1,000 person-years)		Adjusted[a] analysis		FDR,	Adjusted[a] analysis		FDR,
	HL	non-HL	HR (95% CI)	*P*	*P*	HR (95% CI)	*P*	*P*
Total	19.38 (18.25-20.57)	13.98(13.01-15.00)	1.17 (1.07 - 1.29)	0.001	0.003	1.11 (1.01 - 1.22)	0.02	0.05
Gender								
Female	17.74 (16.10-19.50)	13.53 (12.10-15.09)	1.13 (0.97 - 1.30)	0.11	0.21	1.18 (1.02 - 1.35)	0.02	0.05
Male	20.62 (19.08-22.25)	14.32 (13.03-15.71)	1.20 (1.06 - 1.36)	0.003	0.008	1.07 (0.95 - 1.21)	0.29	0.42
Age group(years)								
45-64	5.46 (4.53-6.53)	2.12 (1.56-2.83)	2.21(1.57 - 3.12)	<0.001	<0.001	1.40 (1.12 - 1.75)	0.004	0.01
65-74	20.00 (18.27-21.86)	15.49 (13.95-17.14)	1.13 (0.99 - 1.30)	0.07	0.13	1.09 (0.94 - 1.27)	0.25	0.41
≥75+	48.20 (44.01-52.68)	38.36 (34.48-42.57)	1.07 (0.93 - 1.24)	0.32	0.46	1.01 (0.88 - 1.15)	0.89	0.93

[a]Adjusted for sex, age, residence, insurance premium, coronary artery disease, chronic obstructive pulmonary disease, cerebrovascular disease, diabetes, chronic kidney disease, hypertension, hyperlipidemia, anxiety, depression, alcohol related illness, head injury, rheumatoid arthritis, asthma, length of hospital stay.

Abbreviation HR: hazard ratio; CI = confidence interval; FDR=False discovery rate HL= Hearing Loss.

© 2019 Liu C-M et al. *JAMA Network Open.*
